# Advancing pharmacogenomic research in US Hmong populations: prevalence of key single nucleotide variations in California Hmong

**DOI:** 10.3389/fphar.2024.1432906

**Published:** 2024-09-24

**Authors:** Boguang Sun, Tou Thao, Kathleen Culhane-Pera, Eric Yang, Mai Yang Thor, Pao Yang, Metta Xiong, Zoua Vang, Robert J. Straka

**Affiliations:** ^1^ Department of Experimental and Clinical Pharmacology, College of Pharmacy, University of Minnesota, Minneapolis, MN, United States; ^2^ SoLaHmo Partnership for Health and Wellness, Community-University Healthcare Center, Minneapolis, MN, United States; ^3^ Hmong Youth and Parents United, Sacramento, CA, United States; ^4^ The Fresno Center, Fresno, CA, United States

**Keywords:** pharmacogenetics, precision medicine, gene frequency, Asian Americans, Hmong, minority health

## Abstract

**Introduction:**

In collaboration with the Minnesota Hmong community, we have previously discovered significant differences in allele frequencies for key Single Nucleotide Variations (SNVs) within Very Important Pharmacogenes (VIPs) between Hmong and East Asians. Recognizing the potential clinical implications of these observed differences, we sought to validate these observations in a Hmong cohort residing in California, the state with the largest Hmong population in the US. Robust validation of these differences would affect motivation for clinicians treating individuals who identify as Hmong to consider pharmacogenomic (PGx) testing as a means to improve clinical decision making when using therapeutic agents in this unique population.

**Method:**

Guided by California Hmong community leaders and utilizing the basic approach of community-based participatory research, demographic, clinical information and a buccal swab was obtained from Hmong adults residing in California. A commercial PGx testing panel was performed on these samples and specific allele frequencies of interest were compared between California and Minnesota Hmong. Allele frequency differences between California Hmong, East Asians and Europeans, were also compared. Return-of-PGx-results and presentations of group data were made to members of the Hmong along with PGx educational sessions to help interpret the observations.

**Results:**

In 118 California Hmong who completed the study, the allele frequencies for SNV’s were similar to previous Minnesota Hmong results. Furthermore, out of the 18 SNVs that were not previously reported in Hmong, allele frequencies were statistically different in 38% (7/18) of SNVs comparing California Hmong to East Asians, and in 77.8% (14/18) SNVs comparing California Hmong to Europeans.

**Conclusion:**

These results validate the original study’s findings that Hmong people living in different US locations have similar allele frequencies for key PGx genes. Further, for many of these PGx genes, their allele frequencies are significantly different compared to either East Asians or Europeans. Clinicians should consider these important differences when prescribing medications for people who identify as Hmong.

## Introduction

The United States (US) National Institutes of Health “All of Us” Research Program encourages inclusion of diverse populations in pharmacogenomics (PGx) research (Murray, 2019). In recent years, the diversity of populations participating in PGx studies has grown in part due to increased engagement with broader East Asian (EA) populations (Lo et al., 2020). However, the participation in PGx studies conducted within the US by minority Southeast (SE) Asian groups such as Hmong, Filipinos, and Vietnamese is still lacking. Studies have found that there are clinically relevant differences in PGx markers such as single nucleotide variations (SNVs) among SE Asian subpopulations (Lo et al., 2020; Pan and Xu, 2020). These SE Asian subpopulations are also historically underserved and underrepresented as a subpopulation within the US healthcare system. Failure to include such subpopulations in research might exacerbate health disparities (Gordon et al., 2019). Therefore, there is a critical need to engage underrepresented SE Asian subpopulations, such as Hmong, in PGx studies.

Hmong people were resettled to the US as refugees from Southeast Asia starting after the Vietnam War in 1975 (Hamilton-Merritt, 1993), with the states of California, Minnesota and Wisconsin currently having the top three highest populations per US 2020 Census (Pfeiffer, 2024). For the past two decades, we have used a community-based participatory research approach (Israel et al., 1998) to partner with Minnesota Hmong community members to conduct PGx-based studies in Hmong populations to help redress disparities in pharmacogenomic research (Culhane-Pera et al., 2017a; Culhane-Pera et al., 2017b; Holzer et al., 2021). In 2016–2017, we enrolled 198 self-identified Hmong people at Minnesota and Wisconsin locations in a Very Important Pharmacogenes (VIPs) study called VIP-Hmong in the Midwest (*VIP-MW*) (Wen et al., 2020). Through such efforts, we discovered that there are significant differences in allele frequencies for SNVs within VIPs for Hmong adults compared to EA and European (EU) populations. These VIPs are important determinants which inform drug selection and dosing decisions. For example, some of the genetic variants affect the metabolism and disposition of several medications including anti-thrombotic (warfarin and clopidogrel), anti-hyperuricemic (allopurinol) and mental health medications (antidepressants and antipsychotics) (Wen et al., 2020; Sun et al., 2021).

These unique findings regarding key differences in allele frequencies within VIPs in the midwestern Hmong compared to EA populations have yet to be validated in Hmong residing in other parts of the US (Sun et al., 2022). As the state with the largest population of Hmong in the US (Pfeiffer, 2024), California (CA) Hmong communities can play a significant role in helping to address this gap in knowledge. Consequently, in this study named VIP-Hmong in California (*VIP-HC*), we aimed to 1) validate if our reported allele frequencies of common SNVs in VIPs identified in the *VIP-MW* study are similar to those found in *VIP-HC*, 2) identify those allele frequencies of SNVs in our *VIP-HC* cohort and their clinical implications and 3) conduct return-of-results (ROR) assessment sessions to engage CA Hmong in genetics research and education. Together, these approaches will strengthen the argument that Hmong represent a unique yet underserved ancestral group that may derive benefits from greater knowledge of how to individualize medication dose and selection.

## Methods

### Recruitment and enrollment

Using a community-based participatory research (CBPR) approach (Israel et al., 1998), we created an academic-community partnership between University of Minnesota researchers (RJS, BS, TT) and Hmong leaders and community researchers (EY, MYT, PY, MX, ZV) from two non-profit Hmong organizations at two cities with high Hmong populations in CA—The Fresno Center (TFC) in Fresno, CA and Hmong Youth and Parents United (HYPU) in Sacramento, CA. The academic-community teams created a culturally and linguistically-appropriate research process where recruitment, consent and enrollment occurred at the two community centers, since their physical spaces were familiar and comfortable to community members. We co-designed advertisements aimed at engaging local members of the Hmong community to participate in this project that would ultimately provide each participant with their personal PGx profile and opportunities for interpretation of group data derived from the community. We created a consent process to adjust to participants’ needs, including a written consent form in English and Hmong, and an oral consent process.

At community events in June 2022 at TFC and July 2022 at HYPU, the academic-community team consented and enrolled self-identified Hmong adults 18–80 years of age whose parents were both Hmong. We excluded participants whose primary addresses were not in California and anyone who may have participated in our previous *VIP-MW* study. The bilingual bicultural Hmong researchers led the consent process with support from academic team members. The University of Minnesota Institutional Review Board approved this research (UMN IRB#STUDY00016002).

### Data collection and PGx sequencing

At these community events, the academic-community team collected participants’ demographic data (age, gender, birth country, years in the US, and education level) and anthropometric data (weight and height) (Table 1). We provided verbal and written instructions in English and Hmong on the buccal swab DNA collection procedure as participants self-collected their DNA samples on site. We mailed the DNA samples to Kailos Genetics (Kailos Genetics, Huntsville, AL, United States), which processed the DNA and performed the PGx panel test. The PGx panel test was conducted using the MiSeq System (Illumina) system with paired end 78 bp reads to sequence selected variants within 42 pharmacogenes. The PGxComplete™ panel from Kailos Genetics was utilized to select and enhance specific areas of the genome. After sequencing, a cloud-based analysis system unique to Kailos Genetics was used to conduct various procedures including sample demultiplexing, quality control, alignment to the genome, identifying variants, and generating a report.

**TABLE 1:** Participant characteristics for California (CA) and Midwest (MW) Hmong.

Characteristics	CA Hmong (N = 121)	MW Hmong (N = 198)
Age in years (range, mean ± SE)	18–81, (37.0 ± 1.5)	18–94, (32.6 ± 1.4)
Male gender, N (%)	40 (33.1%)	77 (38.9%)
Height in inches (range, mean ± SE)	54.0–70.0, (61.9 ± 0.3)	53.8–70.0, (62.1 ± 0.3)
Weight in Ib (range, mean ± SE)	90.0–253.2, (152.1 ± 3.0)	82.0–288.0, (148.5 ± 2.3)
BMI in kg/m2 (range, mean ± SE)	18.2–47.9, (27.9 ± 0.5)	16.3–53.8, (27.1 ± 0.4)
Birth country		
United States, N (%)	64 (52.9%)	115 (58.4%)
Laos, N (%)	26 (21.5%)	50 (25.4%)
Thailand, N (%)	31 (25.6%)	32 (16.2%)
Years in the US (range, mean ± SE)	3–45, (28.9 ± 1.0)	1–45, (27.3 ± 1.2)
Highest education (N = 121)		
None, N (%)	20 (16.5%)	20 (11.4%)
High school, N (%)	17 (14.0%)	12 (6.9%)
Technical school, N (%)	2 (1.7%)	4 (4.2%)
College or higher, N (%)	82 (67.8%)	128 (73.1%)

Allele frequency comparisons between CA and MW Hmong.

### Genotype, diplotype, phenotype comparisons and clinical recommendations

For aim 1, we compared the allele frequencies of 21 SNVs generated from the *VIP-HC* cohort and compared those to the previously analyzed *VIP-MW* cohort (Table 2). For aim 2, we conducted additional comparisons for those SNVs whose prevalence proved to be different in Hmong vs. EA’s, as defined in the “PharmGKB Biogeographical Groups” (www.pharmgkb.org/page/biogeographicalGroups) as follows:

1) Allele frequency distributions for an additional 18 SNVs (Table 3) that were not previously reported in *VIP-MW* were compared with EA and EU populations. We chose these 18 SNVs because they were 1) included in the Kailos PGx testing panel; 2) identified within Pharmacogenomics Knowledge Base (PharmGKB) as either Level 1A clinical annotations (www.pharmgkb.org) or those that represented commonly studied alleles among the PharmGKB Tier 1 VIP variants; 3) reported to have a minor allele frequency greater than 1% in either of the CA Hmong, EA and EU cohort; and 4) identified to not deviate from Hardy-Weinberg Equilibrium (HWE) within the CA Hmong sample cohort.
2) Furthermore, diplotype and phenotype distributions (Table 4) were compared between Hmong, EA and EU populations. We chose to report these diplotypes and phenotypes to add to the literature since they were not previously reported in our *VIP-MW* study and they represented statistically significant differences when compared between our combined CA and Midwest Hmong cohorts and other populations. The combined CA and Midwest Hmong cohorts (combined Hmong cohort) was used only when both diplotype and phenotype information were available for both cohorts.
3) To impart some relevance to clinical recommendations, examples of clinically relevant recommendations were constructed. These were informed by the imputed phenotypes represented in Table 5. To construct this table, we assigned an individual having an actionable phenotype when the corresponding clinical recommendation would result in either an adjustment of recommended dose or use of an alternative medication according to specific Clinical Pharmacogenetics Implementation Consortium (CPIC^®^) guidelines applicable to that medication. We only reported phenotypes/clinical recommendations pairs not previously reported in our *VIP-MW* study. For example, as the updated 2022 statin CPIC guidelines currently utilize a combinatorial approach incorporating SNVs within *CYP2C9*, *SLCO1B1* and *ABCG2*, we generated statin PGx-based recommendations for the EA and EU cohorts using simulations of 10,000 EA and 10,000 EU patients based on their reported diplotype frequencies documented in the CPIC gene frequency tables (Cooper-DeHoff et al., 2022). Allele frequencies of the SNVs for EA and EU cohorts were obtained from the 1,000 genome project Phase III data as summarized in the PharmGKB (www.pharmgkb.org). Using the specific gene tables from CPIC website (www.cpicpgx.org), we assigned the diplotypes/phenotypes and associated clinical recommendations for the CA Hmong cohort and extracted sample sizes, diplotypes/phenotypes frequencies for the EA and EU cohorts.

**TABLE 2:** Comparison of allele frequencies between California (CA) and Midwest (MW) Hmong.

Genes	dbSNP number	Nucleotide change	Enzyme activities	CA Hmong frequency (%) (N = 118)^a^	MW Hmong Frequency (%) (N = 198)*
*CYP2C9*	rs1799853	c. 3608C>T	Decreased	0	0
	rs1057910	c. 42614A>C	Decreased	14.8	16.6
*CYP2C19*	rs4244285	c. 19154G>A	None	40.7	42.2
	rs4986893	c. 17948G>A	None	0.4	0.3
	rs28399504	c. 1A>G	None	0	0
	rs56337013	c. 90033C>T	None	0	0
	rs72552267	c. 12748G>A	None	0	0
	rs41291556	c. 12711T>C	None	0	0
	rs12248560	c. −806C>T	Increased	0	0
*CYP4F2*	rs2108622	c. 1297G>A	Decreased	N/A	6.5
*DPYD*	rs3918290	c. 1905 + 1G>A	None	0	0
	rs55886062	c. 1679T>G	None	0	0
*G6PD*	rs1050828	c. 202G>A	Deficient	0	0
	rs5030868	c. 563C>T	Deficient	0	0
	rs72554665	c. 1376G>T/C	Deficient	0	0
*SLCO1B1*	rs4149056	c. 521T>C	Decreased	3.4	3.6
*TPMT*	rs1800462	c. 238G>C	None	0	0
	rs1800460	c. 460G>A	None	0	0
	rs1142345	c. 719A>G	None	0	0.6
	rs1800584	c. 626-1G>A	None	0	0
*VKORC1*	rs992 3231	c. −1639G>A	Decreased	91.1	88.1

^a^ Genotyping results only available in 118/121 CA Hmong participants.

*p values insignificant for all the comparisons.

Allele frequency, Diplotype/Phenotype distributions among Hmong, EA and EU.

**TABLE 3:** Comparison of allele frequencies between California (CA) Hmong, East Asians (EA) and Europeans (EU).

Gene	dbSNP number	CA Hmong frequency (%)	EA frequency (%)	*p* values (CA Hmong vs. EA)	EU frequency (%)	*p* values (CA Hmong vs. EU)
*ABCB1*	rs1045642 G>A	29.7	39.9	**0.00122**	51.8	**<0.0001**
*ABCG2*	rs2231142	0.27.5	29.0	0.57737	9	**<0.0001**
*ADRB1*	rs1801252	19.1	14.1	0.03838	12.7	0.00543
*COMT*	rs4680 G>A	11.0	28.0	**<0.0001**	50	**<0.0001**
*CYP2D6*	rs769258	0	0.3	0.81536	5.3	**0.00052**
*CYP2D6*	rs1065852 G>A	56.8	57.1	0.96489	20.2	**<0.0001**
*CYP2D6*	rs16947 G>A	5.5	14	**0.00027**	34.3	**<0.0001**
*CYP2D6*	rs28371725	1.7	3.8	0.06685	9.3	**<0.0001**
*CYP2D6*	rs3892097	0	0.2	1	18.6	**<0.0001**
*CYP3A4*	rs2242480	39.4	26.8	**<0.0001**	8.2	**<0.0001**
*CYP3A4*	rs35599367	0	0	1	5	**0.00079**
*CYP3A5*	rs776746 C>T	51.3	28.7	**<0.0001**	5.7	**<0.0001**
*DPYD*	rs17376848	12.3	3.7	**<0.0001**	11.4	0.75233
*DPYD*	rs1801159	26.3	26.6	0.85660	18.8	0.00804
*DPYD*	rs1801265	13.6	8.6	0.01130	21.5	0.00432
*DPYD*	rs2297595	0.4	1.8	0.06841	11.9	**<0.0001**
*MTHFR*	rs1801133	19.1	29.6	**0.00062**	36.5	**<0.0001**
*SLCO1B1*	rs2306283 A>G	67.8	76.2	0.00361	40.3	**<0.0001**

Bold denotes significant differences after bonferroni correction.

**TABLE 4:** Comparison of diplotypes and phenotypes frequency between Hmong, East Asians (EA) and Europeans (EU).

Genes	Phenotype (with examples of diplotypes	Hmong count	Hmong, frequency (%)	EA frequency (%)	*p* values (CA Hmong vs. EA)	EU frequency (%)	*p* values (CA Hmong vs. EA)
*ABCG2*	Normal function (GG)	61	51.7	48	0.541	80.3	**<0.0001**
	Decreased function (GT)	49	41.5	42.6		18.6	
	Poor function (TT)	8	6.8	9.4		1.1	
*CYP3A5*	Normal metabolizer (*1/*1)	31	26.2	6.4	**<0.0001**	0.5	**<0.0001**
	Intermediate metabolizer (*1/*3)	59	50	37.9		13.7	
	Poor metabolizer (*3/*3)	28	23.7	55.7		85.7	
*CYP2C9*	Normal (*1/*1) or AS = 2	214	68.8	83.8	**<0.0001**	62.8	0.015
	Intermediate metabolizer (*1/*2) or AS* = 1.5	0	0	5.3		20.8	
	Intermediate metabolizer (*1/*3) or AS = 1.0	95	30.5	9.9		13.8	
	Poor metabolizer (*2/*3, *3/*3) or AS = 0.5	2	0.6	1		2.6	
*CYP2C19*	Normal (*1/*1)	99	34.3	38.1	**0.00092**	39.6	**<0.0001**
	Intermediate metabolizer (*1/*2, *1/*3)	135	46.9	45.9		26.1	
	Poor metabolizer (*2/*2, *2/*3, *3/*3)	54	18.8	13		2.4	
	Ultrarapid metabolizer (*17/*17)	0	0	0		4.6	
	Rapid metabolizer (*1/*17)	0	0	3		27.1	
*SLCO1B1*	Normal function (*1/*1,*1/*37,*37/*37	111	94.1	75.7	**<0.0001**	65.6	**<0.0001**
	Decreased function (*1/*15, *15/*37)	7	5.9	21.8		28.3	
	Poor function (*15/*15)	0	0	1.6		2.9	
	Increased function (*20/*20)	0	0	0		3	
*VKORC1*	Normal activity	7	2.3	1.8	0.593	34.4	**<0.0001**
	Decreased activity (carry at least one rs9923231 variant	288	97.6	98.2		65.6	

*AS is activity score as defined in the CPIC guidelines (Caudle et al., 2014).

Bold indicates statistical significance.

**TABLE 5:** Comparison of percentage of participants with actionable medication recommendation based on phenotypes between Hmong, East Asians (EA) and Europeans (EU).

Medications	Genes	Hmong count	Hmong, frequency (%)	EA, frequency (%)	*p* values (CA Hmong vs. EA	EU, frequency (%)	*p* values (CA Hmong vs. EU)
Simvastatin	*SLCO1B1*	8	6.1	23.6	**<0.0001**	31.2	**<0.0001**
Fluvastatin	*CYP2C9*, *SLCO1B1*	33	28.2	17.5	**0.00304**	39.1	0.013737
Rosuvastatin	*ABCG2*, *SLCO1B1*	8	6.8	10.9	0.152724	4.2	0.121464
Tacrolimus	*CYP3A5*	90	76.1	44.3	**<0.0001**	14.1	**<0.0001**
Clopidogrel	*CYP2C19*	189	66.1	58.9	0.016	28.2	**<0.0001**
NSAIDs^a^	*CYP2C9*	97	31.2	10.9	**<0.0001**	16.4	**<0.0001**
Phenytoin	*CYP2C9*	97	31.2	10.9	**<0.0001**	16.4	**<0.0001**

^a^ Actionable phenotypes are those with an activity score of 1 or lower.

Bold indicates statistical significance.

### Return of PGx results to CA Hmong participants

After the completion of PGx data assembly and analysis, the academic-community team co-created a ROR group report to send to participants (appendix). We focused on readability, comprehensibility and comprehensiveness to inform participants on how to understand PGx information and on how the unique genetic profile of a Hmong individual may translate into important clinical implications. The ROR group report along with the individualized full Kailos PGx report was mailed to each participant individually, about 6–7 months after final enrollment. Shortly after the mailings, two group information sessions were held via live interactive video call to gather participants’ reactions to the PGx reports and provide an opportunity for participants to ask questions regarding general interpretations of the group results. We later shared the recordings of the ROR sessions with all participants regardless of their direct participation in live sessions. A 12-question survey focused on our Hmong participants’ attitude and knowledge about PGx testing was sent to all participants. The survey was based on the validated Minnesota Assessment of Pharmacogenomic Literacy survey (MAPL) (Allen et al., 2022), was modified to include specific questions related to our study and was translated into Hmong (Allen et al., 2022) (Supplementary Table S1).

### Statistical analysis

We summarized baseline characteristics using descriptive statistics. Chi-squared test with one degree of freedom was used to test the departure from HWE for each allele in the CA Hmong cohort. A chi-squared test with Yates’ continuity correction or Fisher exact test was used for allele, diplotype/phenotype and clinical recommendation frequency comparisons. Type I error rate was adjusted using a Bonferroni correction for multiple comparisons. All data analyses were performed with the use of R (v4.2.2, www.R-project.org).

## Results

### Participant characteristics

At two community enrollment events in Fresno and Sacramento, CA, we consented and recruited 121 Hmong adult participants who provided a buccal swab for DNA collection and responded to our baseline data collection survey. Table 1 provides general patient characteristics comparing this *VIP-HC* cohort and previous *VIP-MW* cohort. Overall, the CA and MW Hmong cohorts were comparable in terms of distributions of age, gender, weight/height, BMI, years in US, birth country, education level.

Overall, 118 out 121 participants in the *VIP-HC* cohort had sufficient DNA samples collected and therefore were included in the final analysis.

Table 2 shows that allele frequencies were not statistically different between CA and Midwest Hmong within the 21 SNVs reported in the previous *VIP-MW* study. Of note, the PGx panel conducted within the CA cohort did not include the rs2108622 in *CYP4F2*.

Table 3 shows the allele frequency comparisons among CA Hmong, EA and EU cohorts. Out of the 18 SNVs within 10 VIPs (*ABCB1*, *ABCG2*, *ADRB1*, *CYP2D6*, *COMT*, *CYP3A4*, *CYP3A5*, *DPYD*, *SLCO1B1* and *MTHFR*), allele frequencies were statistically different (*p* < 0.002) in 38% (7/18) of SNVs between CA Hmong and EA, and in 77.8% (14/18) of SNVs between CA Hmong and EU. Specifically, comparing CA Hmong and EA, these following allele frequencies were statistically different: *ABCB1*-rs1045642 (29.7% vs. 39.9%), *COMT*-rs4680 (11% vs. 28%), *CYP2D6*-rs16947 (5.5% vs. 14%), *CYP3A4*-rs2242480 (39.4% vs. 26.8%), *CYP3A5*-rs776746 (51.3% vs. 28.7%), *DPYD*-rs17376848 (12.3% vs. 3.7%), *MTHFR*-rs1801133 (19.1% vs. 29.6%).

Table 4 shows the diplotype/phenotype distribution comparisons among Hmong, EA and EU cohorts. Out of the 6 VIPs including *ABCG2*, *CYP3A5*, *CYP2C9*, *CYP2C19*, *SLCO1B1* and *VKORC1*, diplotype/phenotype distributions were statistically different (*p* < 0.008) in 66.7% (4/6) VIPs between Hmong and EA in *CYP3A5*, *CYP2C9*, *CYP2C19*, *SLCO1B1* and in 83.3% (5/6) VIPs between Hmong and EU in *ABCG2*, *CYP3A5*, *CYP2C19*, *SLCO1B1* and *VKORC1*.

Table 5 shows frequency comparisons of individuals having actionable phenotypes among Hmong, EA, EU, in medications including simvastatin, fluvastatin, rosuvastatin, tacrolimus, clopidogrel, NSAIDs, and phenytoin. Out of the 7 medications included, the percentage of individuals having actionable phenotypes were statistically different (*p* < 0.007) in 71.4% (5/7) medications between Hmong and EA, and in 71.4% (5/7) medications between Hmong and EU. Specifically, comparing Hmong vs. EA, 6% vs. 24% had actionable phenotypes within *SLCO1B1* for simvastatin, 28% vs. 18% for fluvastatin (determined by combination of *CYP2C9* and *SLCO1B1* phenotypes), 76% vs. 44% for tacrolimus (determined by *CYP3A5* phenotypes), and 32% vs. 11% for both NSAIDs and phenytoin (determined by *CYP2C9* phenotypes).

### Return of results in the CA Hmong community

Overall, 12.4% (15/121) participants attended the ROR group session (The Fresno Center in-person (N = 6), HYPU in person (N = 4), Zoom (N = 5). General themes relating to comments received from our Hmong participants implied that their perception of PGx research was that such information and knowledge would help the Hmong community to build their unique identity and assist the Hmong to have greater leverage for individualizing selection of medications and dosages that are more suitable for Hmong individuals. Overall, 19.8% (24/121) participants completed the PGx written survey. Most participants (>95%) had a positive attitude toward PGx in enhancing medical care and research. 20.8% (5/24) of participants expressed concerns over the privacy of their PGx data. In general, participants agreed that the ROR session and materials were helpful and educational for them to understand PGx in general and to appreciate Hmong’s uniqueness regarding PGx findings including their clinical and translational significance. The detailed summary of survey responses is attached in Supplementary Table S2.

## Discussion

To our knowledge, this *VIP-HC* study represents the first PGx-based research study that specifically engaged individuals who identify as Hmong residing in CA. As such, within a state with over 1,10,000 Hmong in the US, we validated the unique allele frequency differences previously observed in the *VIP-MW* study using a CA Hmong cohort. Additionally, we identified allele frequency differences in select SNVs of VIPs that have translational and clinical relevance according to contemporary CPIC guidelines. Finally, engagement of our community colleagues and the ensuing collaborative work that was done to design and disseminate our group ROR report to community members helped advance our collective goals. Specifically, by engaging members of the Hmong community in research that recognizes their unique identity and potential differences in responses to medications, we feel this helped the community engage in this rapidly advancing field of translational science.

Using the Kailos PGx testing panel for our CA Hmong cohort enabled us to report and compare frequencies of 18 SNVs that were not previously reported in Hmong. We found that 38% (7/18) of these SNVs were significantly different between CA Hmong and EA, whereas 77.8% (14/18) were significantly different between CA Hmong and EU. One practical translational application of our findings may be represented by the following scenario based on CPIC guidelines (Caudle et al., 2014). The allele frequency of rs776746 within CYP3A5 was significantly higher in the CA Hmong cohort compared to either EA or EU populations (51.3% vs. 28.7% vs. 5.7% respectively). Our findings based on genotype alone would suggest that 76% of CA Hmong would likely require upward adjustments to their tacrolimus starting dose, in comparison to 44% of EA and only 15% of EU individuals based on the CPIC guidelines (Birdwell et al., 2015). Studies have shown that underserved and underrepresented populations, such as Hmong, have a higher risk of end-stage renal failure and subsequent kidney transplantation where tacrolimus’s usage is prevalent (Ward, 2008; Nicholas et al., 2015). Therefore, awareness of this fact and the potential application of PGx-guided tacrolimus dosing applied to Hmong requiring organ transplants, represents a unique opportunity to achieve better treatment outcomes. Although a proof of concept study is needed, such findings may represent one practical application which could, in part, help address existing health disparities in outcomes.

Due to the pilot nature of the previous *VIP-MW* study, we only included a limited selection of SNVs. Consequently, we have a limited ability to comprehensively call certain haplotypes for several VIPs in that population. One example includes calls for *SLCO1B1* haplotypes. In the previous *VIP-MW* study, we assigned *5 haplotype to participants based only on one variant (rs4149056) while the more contemporary definition of *5 requires testing of at least one other variant (rs2306283) (Ramsey et al., 2023). With the expanded testing panel provided by Kailos, we were able to update the *SLCO1B1* haplotype calls to include more precise haplotypes such as *15 and *37. As such, we can more specifically report that 94% of the CA Hmong have normal functioning *SLCO1B1*(*1/*1, *1/*37 or *37/*37) compared to 76% and 66% in EA and EU populations, respectively.

With the updates of CPIC guidelines pertaining to statins (Cooper-DeHoff et al., 2022), we were able to generate additional CPIC clinical recommendations based on the phenotype calls for the CA Hmong cohort in comparison with EA and EU populations. Only 6% of Hmong have actionable *SLCO1B1* phenotypes related to simvastatin compared to 24% and 31% in EA and EU, respectively. More Hmong (32%) have actionable *CYP2C9* phenotypes which are relevant to NSAIDs and fluvastatin than EA (11%) and EU (16%). Furthermore, when combining *SLCO1B1* and *CYP2C9* phenotypes, 28% Hmong have actionable combined phenotypes compared to 18% and 39% in EA and EU, respectively, when selecting fluvastatin dosing according to the latest CPIC guidelines for statins (Cooper-DeHoff et al., 2022). These differences underscore the importance of acquiring individual PGx test results rather than projecting assumptions about the impact of PGx based on presumptive, inadequately granular categorizations of individuals assuming an ancestral affiliation.

Our study has several limitations. As the participants were a volunteer sample, it may be argued that the results have limited generalizability. However, since the results in this CA cohort confirmed the results previously found in the Midwest cohort, the results give some support to their generalizability. Secondly, we did not report allele frequencies related to *CYP2D6* because the Kailos PGx panel, at that time, had yet to include structural variants for *CYP2D6*. However, we have previously reported novel genetic variants pertaining to *CYP2D6* found in the Hmong (Wen et al., 2022). Thirdly, only 12.4% (15/121) of the participants attended our ROR session and 19.8% (24/121) answered the PGx survey. Although engagement of underrepresented populations in clinical research is well recognized to be limited, this observation foreshadows challenges and the need for more effective tools to achieve our ultimate goal of improving PGx education and dissemination of findings to members of such communities. In an attempt to remedy the challenges of live attendance, we shared our ROR session recording to our participants and offered opportunities to provide additional counseling, should participants have further inquiries.

Knowledge of differences in allele frequencies within VIPs between Hmong and other populations have important clinical implications to addressing health disparities. For example, in a pilot retrospective cohort study, we observed that Hmong required lower mean warfarin stable doses compared to other East Asians in part due to Hmong’s higher CYP2C9*3 frequency (Sun et al., 2021). Such examples illustrate the potential translational significance of our findings. Consequently, we believe it is critical that future studies investigate PGx information using participants from diverse populations and subpopulations. Individualization of drug dosage and selection using PGx information can help inform clinicians as to the optimal use of medications for Hmong patients in clinical settings. This approach could help further promote precision medicine and reduce health disparities in the Hmong and ultimately other underrepresented communities.

## Conclusion

By partnering and collaborating with key leaders within the Hmong CA community, we were successful in engaging members of the Hmong communities in two locations within California who have previously not participated in PGx studies. We validated unique allele frequencies of SNVs previously found in the VIP-MW study and discovered several differential allele frequencies within VIPs that could have clinical implications for select gene/drug pairs such as *CYP3A5*/tacrolimus and other cases. We also communicated our findings and provided PGx education in ROR sessions. Our study strengthened the generalizability of several genetic variants displaying unique frequencies within those who identify as having Hmong ancestry within the US and, in doing so, have demonstrated several potentially important clinical implications of considering PGx testing for this population.

## Data Availability

The datasets presented in this article are not readily available because the participants had the options on their consent forms to select to “not release genetic information beyond the scope of this study”, and because of HIPAA requirements for all participants. Requests to access the datasets should be directed to corresponding author.
