# Supplementary material for: Advancing pharmacogenomic research in US Hmong populations: prevalence of key single nucleotide variations in California Hmong
Source: Front Pharmacol. 2024 Sep 24;15:1432906. doi: 10.3389/fphar.2024.1432906 (PMC11462547; doi:10.3389/fphar.2024.1432906)
Supplement: Supplementary file 1 [file Table1.DOCX]

**Table S1** Pharmacogenomics in California Hmong Post Study Survey Questions

| 12 | (**Answer this only if you attended the group sessions**) Please rate the following statement: I think the group sessions were helpful for me to understand more about pharmacogenomics in general and in Hmong. | Strongly agree | Agree | Do not agree or disagree | Disagree | Strongly disagree |  |  |
| --- | --- | --- | --- | --- | --- | --- | --- | --- |
| 11 | Please rate the following statement: I think the pharmacogenomics group report is helpful for me to appreciate Hmong’s uniqueness in genetics and how that would impact medication uses for Hmong. | Strongly agree | Agree | Do not agree or disagree | Disagree | Strongly disagree |  |  |
| 10 | Please rate the following statement: I think the individual pharmacogenomics report I received is helpful for me and my healthcare providers to select the best medications and doses. | Strongly agree | Agree | Do not agree or disagree | Disagree | Strongly disagree |  |  |
| 9 | Please rate the following statement: I would value a safe and private way to share my own information with researchers so they can study how pharmacogenomics influences the safety and effectiveness of medications in the Hmong. | Strongly agree | Agree | Do not agree or disagree | Disagree | Strongly disagree |  |  |
| 8 | Please rate the following statement: I believe we all can benefit if we share our pharmacogenomic data for research. | Strongly agree | Agree | Do not agree or disagree | Disagree | Strongly disagree |  |  |
| 7 | Please rate the following statement: I would trust genetic companies to keep my pharmacogenomic data private. | Strongly agree | Agree | Do not agree or disagree | Disagree | Strongly disagree |  |  |
| 6 | Please rate the following statement: I would trust medical researchers to keep my pharmacogenomic data private. | Strongly agree | Agree | Do not agree or disagree | Disagree | Strongly disagree |  |  |
| 5 | Please rate the following statement: I would trust my healthcare professionals to keep my pharmacogenomic data private. | Strongly agree | Agree | Do not agree or disagree | Disagree | Strongly disagree |  |  |
| 4 | Please rate the following statement: I worry about the privacy of my pharmacogenomic data. | Strongly agree | Agree | Do not agree or disagree | Disagree | Strongly disagree |  |  |
| 3 | If the option were available to me, the amount I would pay out-of-pocket for pharmacogenomic test would be: | I would not pay any out-of-pocket money | $0-$49 | $50-$149 | $150-$249 | $250-$499 | $500-$999 | $1000+ |
| 2 | Please rate the following statement: I would be comfortable getting a pharmacogenomic test from a direct-to-consumer company or at a pharmacy that was not ordered by my physician or other healthcare providers. | Strongly agree | Agree | Do not agree or disagree | Disagree | Strongly disagree |  |  |
| 1 | Please rate the following statement: I would feel comfortable getting a pharmacogenomic test if recommended by a healthcare provider. | Strongly agree | Agree | Do not agree or disagree | Disagree | Strongly disagree |  |  |
| Question Number | Question Text | Options | | | | | | |

**Table S2** Pharmacogenomics in California Hmong Post Study Survey Response Summary

| Question number | Strongly agree or agree, n (%) | Do not agree or disagree, n (%) | Disagree or strongly disagree, n (%) |  |
| --- | --- | --- | --- | --- |
| 1 | 24 (100) | 0 (0) | 0 (0) |  |
| 2 | 18 (75) | 3 (12.5) | 3 (12.5) |  |
| 4 | 16 (66.7) | 3 (12.5) | 5 (20.8) |  |
| 5 | 22 (91.7) | 2 (8.3) | 0 (0) |  |
| 6 | 22 (91.7) | 2 (8.3) | 0 (0) |  |
| 7 | 19 (79.2) | 3 (12.5) | 2 (8.3) |  |
| 8 | 22 (91.7) | 2 (8.3) | 0 (0) |  |
| 9 | 23 (95.8) | 0 (0) | 1 (4.2) |  |
| 10 | 23 (95.8) | 1 (4.2) | 0 (0) |  |
| 11 | 23 (95.8) | 1 (4.2) | 0 (0) |  |
| 12 | 6 (100) | 0 (0) | 0 (0) |  |
|  | “Will not pay”, n (%) | $0-$49, n (%) | $50-$149, n (%) | >$150, n (%) |
| 3 | 6 (25) | 13 (54.2) | 5 (20.8) | 0 (0) |
