# Supplementary material for: Advancing pharmacogenomic research in US Hmong populations: prevalence of key single nucleotide variations in California Hmong
Source: Front Pharmacol. 2024 Sep 24;15:1432906. doi: 10.3389/fphar.2024.1432906 (PMC11462547; doi:10.3389/fphar.2024.1432906)
Supplement: Supplementary file 2 [file DataSheet1.PDF]

UNIVERSITY OF MINNESOTA

Twin Cities Campus

Department of Experimental and Clinical Pharmacology
College of Pharmacy

7-115 Weaver-Densford Hall
308 Harvard Street S.E.
Minneapolis, MN 55455
Office: 612-625-2160

Dear Participant:

Thank you for being in the **"Very Important Pharmacogenes for the Hmong in California" Research Study**, also called *VIP-Hmong California (VIP-HC) Study.*

We are excited to share the results of the study with you:

- **Page 2**: Summary of **VIP-HC Research Study.**
- **Page 3**: We show one example how a person's genes can change a medicine's effects. This example is about clopidogrel (Plavix) which is a blood thinner that can prevent clots, when used for people with heart disease, heart attacks and strokes.
- **Page 4**: **Group results of key gene variants comparing** Hmong in California (CA) with Hmong in Minnesota (MN), East Asians (Chinese, Japanese, and Koreans), and Europeans from other studies.
- **Your personal pharmacogenomics testing report about your genetic/DNA results** provided by Kailos Genetics.

You can discuss your results with doctors or pharmacists who may know about pharmacogenomics and its possible effects on your present or future medicines. Remember, these study's results are for **"research/educational purposes only"** and are not for direct patient care. You **should not change your medicine** without talking with your doctor and/or pharmacist first. They can discuss these results with you and may recommend more tests or repeat this test.

We hope you can come to a group zoom session. We will talk about the group results, and about your personal results if you want. We will send a zoom link to a group discussion/video chat session later.

Thank you,

Robert J. Straka (strak001@umn.edu)

The VIP-HC Research Team:

Robert J. Straka Pharm.D. FCCP, Professor, University of Minnesota

Boguang Sun, Pharm.D., Research Assistant, University of Minnesota

Kathie Culhane-Pera MD, MA, Family Medicine Doctor, University of Minnesota

Tou Thao, Research Assistant, University of Minnesota

The Fresno Center, Fresno, California

Hmong Youth and Parents United, Sacramento, California

Kailos Genetics, Huntsville, Alabama (In-kind provision of pharmacogenomics reports)

Useful website for further pharmacogenomics information:

cpicpgx.org/guidelines/

www.pharmgkb.org/

UNIVERSITY OF MINNESOTA

Twin Cities Campus

Department of Experimental and Clinical Pharmacology
College of Pharmacy

7-115 Weaver-Densford Hall
308 Harvard Street S.E.
Minneapolis, MN 55455
Office: 612-625-2160

The VIP-HC Research Study Summary

What did we do?

Team: We are a partnership between the Hmong community in Fresno (The Fresno Center) and in Sacramento (Hmong Youth and Parents United), academic researchers at the University of Minnesota, and Kailos Genetics. Together, we designed the study and the community plan for engagement.

Purpose: Our previous Minnesota-based study showed that Hmong have different variations of certain genes (DNA) compared to East Asians (Chinese, Japanese and Koreans), which may affect some medicines. These genes are called *Very Important Pharmacogenes or VIPs*. This current study explores if such differences are also present in Hmong *living in California*.

Methods: In 2022, 121 members of the Hmong community joined the study, answered a survey and did a cheek-swab for DNA/genes. Kailos Genetics analyzed the cheek samples for DNA/genes. We looked for 215 variations of certain genes that are important for over 50 medicines in 118 people.

Interpret test results: From various published studies and guidelines, we looked to see if these genetic variations would change recommendations about using some medicines.

- If people **do not** have genetic variations affecting some medicines, they could take the usual starting dose of the medicine.
- If people do have genetic variations, they may be guided to start the medicine at a lower dose or a higher dose or take a different medicine altogether.

What did we find?

- We found the prevalence of genetic variations within key genes are **similar between Hmong in California and Hmong in Minnesota.**
- In general, Hmong have **differences in the prevalence of genetic variations compared to other populations, such as East Asians and Europeans.**

What can you do with this information?

- You can **share this information with your doctor or pharmacist**, who may know about such reports and your specific medicines.
- If you are taking medicines that are affected by your pharmacogenomics results, you and your doctor or pharmacist should talk about what it *may mean for you, if confirmed by additional testing*. You **should not change your medicine** without talking with your doctor or pharmacist first. Your doctor or pharmacist may repeat the pharmacogenetic test. Your doctor or pharmacist may or may not change the dose, change the medicine, or consult with others.
- You and your doctor or pharmacist **may save the results for future use** when you could talk about the right medicines and the right doses for you as your medication needs change in the future.

UNIVERSITY OF MINNESOTA

Twin Cities Campus

Department of Experimental and Clinical Pharmacology

College of Pharmacy

7-115 Weaver-Densford Hall
308 Harvard Street S.E.
Minneapolis, MN 55455

Office: 612-625-2160

Figure 1: A Demonstration of How Different Gene/DNA Patterns Between Hmong and Europeans Result in Different Drug Effect

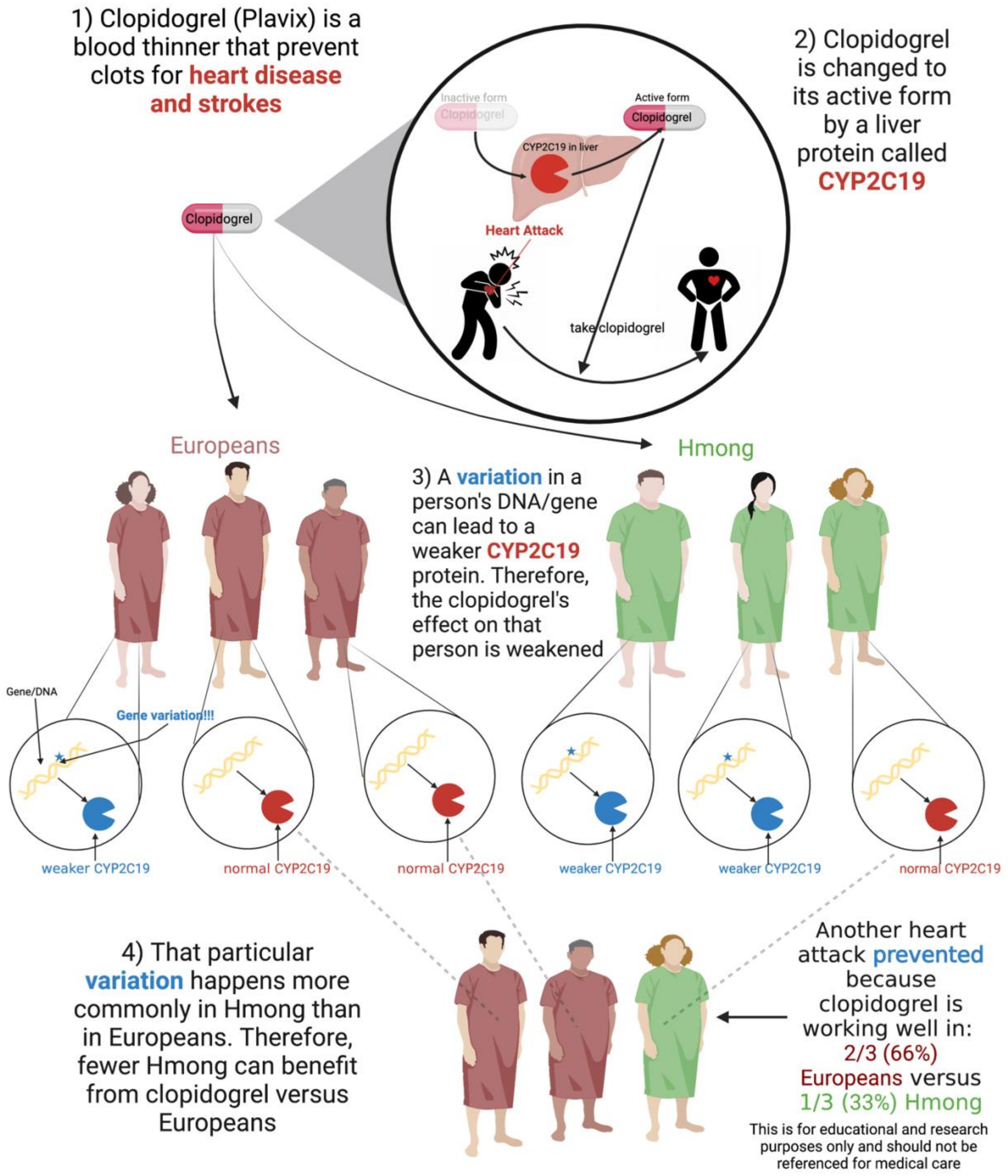

UNIVERSITY OF MINNESOTA

Twin Cities Campus

Department of Experimental and Clinical Pharmacology
College of Pharmacy

7-115 Weaver-Densford Hall
308 Harvard Street S.E.
Minneapolis, MN 55455
Office: 612-625-2160

Figure 2: Comparing Some* Group Results of Gene Variants for California (CA) Hmong, Minnesota (MN) Hmong, East Asians, and Europeans

Medicine and Use	Gene/Variant Pair	Prevalence of Variant in a Population (For example: a prevalence of 0.39 means 39% of the population have that gene variant)	Population
Warfarin/ Coumadin: Blood thinner to prevent clots	VKORC1-rs9923231	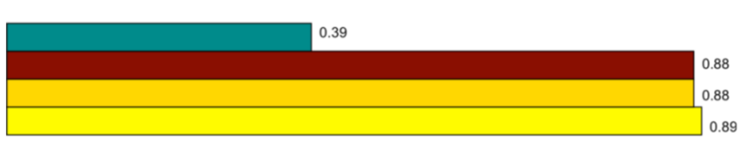	European, East Asian, MN Hmong, CA Hmong
	CYP2C9-rs1057910	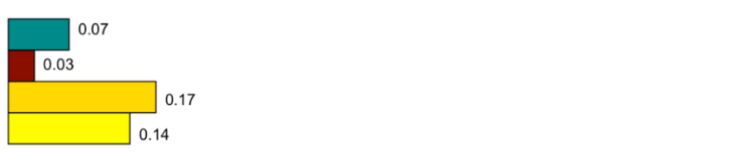	European, East Asian, MN Hmong, CA Hmong
Statins: Cholesterol lowering drugs	SLCO1B1-rs4149056	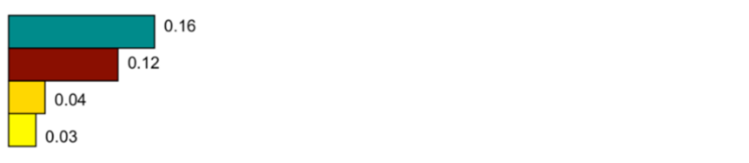	European, East Asian, MN Hmong, CA Hmong
	CYP2C9-rs1057910	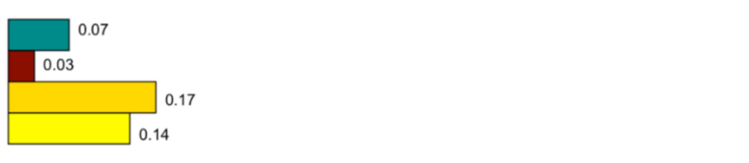	European, East Asian, MN Hmong, CA Hmong
	ABCG2-rs2231142	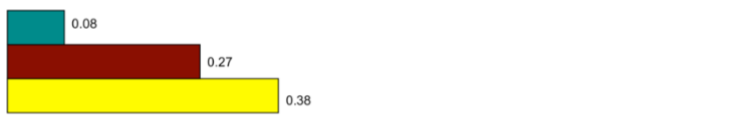	European, East Asian, CA Hmong
Clopidogrel/Plavix: Blood thinner to prevent clots	CYP2C19-rs4986893	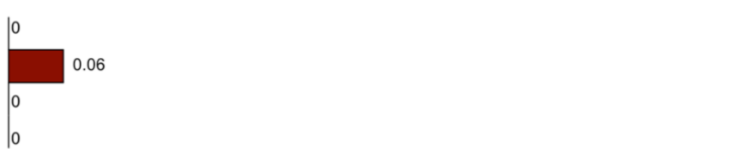	European, East Asian, MN Hmong, CA Hmong
	CYP2C19-rs4244285	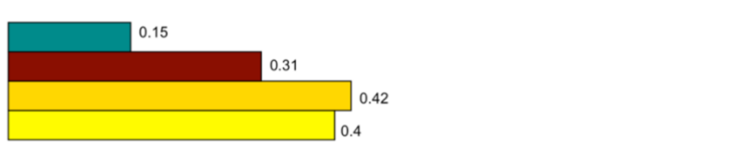	European, East Asian, MN Hmong, CA Hmong
Tacrolimus: Immunosuppressant used in organ transplants	CYP3A4-rs2242480	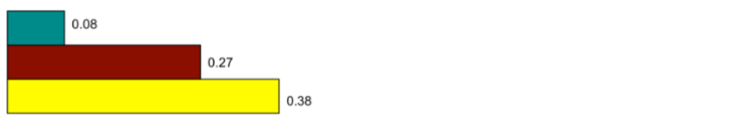	European, East Asian, CA Hmong
	CYP3A5-rs2231142	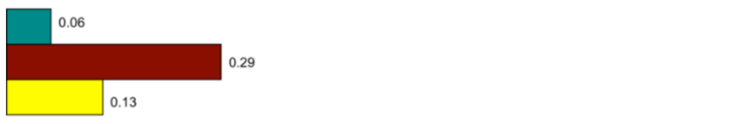	European, East Asian, CA Hmong

*For a more comprehensive result, please refer to your individual pharmacogenomics report.
